# Barriers and Challenges to Implementing Whole Blood Transfusion Protocols in Civilian Hospitals: A Systematic Review and Meta-Analysis

**DOI:** 10.3390/jcm13164726

**Published:** 2024-08-12

**Authors:** Thamer Nouh, Mishary Shalhoub, Ahmed Alburakan, Nawaf Alshahwan, Lama Alzelfawi, Ebtesam Almajed, Zeena Alhindawi, Rawan Bin Salamah, Wijdan AlMutiri, Ebtisam Alruwaili, Abdulelah Alhawas, Nourah Almutairi, Hassan Mashbari

**Affiliations:** 1 Trauma and Acute Care Department, King Saud University, Riyadh 12271, Saudi Arabia; tnouh@ksu.edu.sa (T.N.); aalburakan@ksu.edu.sa (A.A.); nalshahwan@ksu.edu.sa (N.A.); 2 Trauma and Acute Care Department, King Abdullah Bin Abdulaziz University Hospital, Riyadh 11564, Saudi Arabia; mish.shalhoub@yahoo.com; 3 College of Medicine, Princess Nourah Bint Abdulrahman University, Riyadh 11564, Saudi Arabia; alzelfawilama@gmail.com (L.A.); 8ebtesamalmajed@gmail.com (E.A.); alizaina74@gmail.com (Z.A.); rawanbinsalamah@gmail.com (R.B.S.); wjal1189@gmail.com (W.A.); 4 College of Medicine, Aljouf University, Sakaka 72388, Saudi Arabia; dr.ebtisamalruwaili@gmail.com; 5 College of Medicine, King Saud Bin Abdulaziz University for Health Sciences, Al-Ahsa 31982, Saudi Arabia; abdulelah.y.hawas@gmail.com; 6 College of Medicine, Majmaah University, Al Majma’ah 15341, Saudi Arabia; 1nourahamdan@gmail.com; 7 College of Medicine, Jazan University, Jazan 45142, Saudi Arabia

**Keywords:** whole blood, transfusion, mortality, civilian hospital, trauma

## Abstract

**Background:** Whole blood is a product that contains all three blood components (plasma, red blood cells, and platelets). This systemic review and meta-analysis was conducted to identify barriers and obstacles to establishing whole blood transfusion protocols in civilian hospitals. **Methods:** The study was conducted using PRISMA guidelines with PROSPERO registration No. CRD42024519898. Traumatic patients who needed or received whole blood transfusion were included. A systematic literature review employed a comprehensive search strategy through the PubMed, Google Scholar, Web of Science, ScienceDirect, and ProQuest databases. Meta-analysis was utilized to analyze the outcomes. The risk of bias was assessed using the Newcastle–Ottawa Scale. **Results:** In total, 310 studies were identified, and 11 studies met the inclusion criteria. The following intervals were used to assess the prevalence of mortality: 6 h 12.15% (0.081, 95% CI [0.023, 0.139]), 24 h 14.08% (0.141, 95% CI [0.111, 0.171]), delayed mortality (28–30 days) 22.89% (0.284, 95% CI [0.207, 0.360]), and in-hospital 18.72%, with relative risk (0.176, 95% CI [0.114,0.238]). **Conclusions:** Traumatic patients can be effectively resuscitated and stabilized with whole blood transfusion (WBT), but it is essential to provide ongoing critical care, address logistical challenges, and prevent blood product wastage. We recommend utilizing WBT in the early stages of resuscitation for adult civilian trauma patients.

## 1. Introduction

In a whole blood (WB) transfusion, all vital components are contained in one product—plasma, red blood cells (RBCs), and platelets—which facilitates the early inclusion of plasma, potentially enhancing the overall effectiveness of transfusion in managing hemorrhage in the trauma setting [1]. Following collection, fresh whole blood (FWB) possesses a shelf life of 24 h at ambient temperature. Alternatively, it can be stored within 8 h of collection through refrigeration for a maximum duration of 35 days. According to the WHO Guidelines and Principles for Safe Blood Transfusion Practice, whole blood must be stored at a temperature range of +2 °C to +6 °C to maintain its viability and oxygen-carrying capacity [2]. In emergency settings where a patient’s blood type is unknown, low-titer group O whole blood (LTOWB) can be a safe and adequate blood product for initial transfusion due to its universal compatibility due to the absence of A, B, and Rh antigens, minimizing the risk of immunological rejection. However, it can be regarded as the gold standard for treating significant bleeding injuries [3].

Early attempts at blood transfusion can be traced back to 1795, with Dr. Philip Syng Physick performing the first documented instance. In 1818, Dr. James Blundell achieved landmark success by administering a human blood transfusion to treat a patient suffering from hemorrhage. This marked a significant step forward, but the widespread use of blood transfusions awaited further scientific advancements [4]. Subsequently, fresh frozen plasma (FFP) and packed RBCs were available during the early years of the Vietnam War (1954–1975) owing to the advances in blood fractionation [5].

WB incorporates all essential blood components into a single unit. Conversely, component therapy necessitates handling multiple blood products with diverse storage protocols, considerably complicating logistical management for healthcare professionals [6]. Furthermore, WB exhibits resuscitative properties in life-threatening bleeding incidents by rapidly restoring oxygen-carrying capacity, coagulation factors, platelets, and overall blood volume [7].

The integration of LTOWB into trauma center protocols presents several significant challenges. Firstly, establishing a dedicated whole blood program within a blood bank requires substantial capital expenditures, including equipment acquisition and robust quality control measures. Secondly, the successful implementation of LTOWB hinges on cold chain management, which is crucial for preserving LTOWB’s viability and functionality. Logistical complexities arise in ensuring proper handling and transportation of LTOWB units, potentially impacting their availability during critical situations. Finally, a significant challenge associated with LTOWB utilization concerns blood product waste, as aligning supply with unpredictable patient demand can lead to the expiration and subsequent discarding of LTOWB units, resulting in wasted resources [8].

While whole blood transfusion offers a life-saving intervention in trauma patients, cultural and religious beliefs can also hinder its application. Notably, a well-defined patient population, such as Jehovah’s Witnesses adhering to specific religious tenets, may decline blood transfusions. They fervently believe that anyone receiving blood transfusions would face judgment from Jehovah. Because they feel that having a transfusion puts them and their children at risk of losing their eternal salvation, Jehovah’s Witnesses routinely refuse to receive transfusions for themselves and their children [9]. This systematic review and meta-analysis aimed to investigate the barriers and obstacles associated with whole blood transfusion in trauma patients in a civilian setting.

## 2. Methods

### 2.1. Study Eligibility Criteria

This systematic review and meta-analysis study followed the ‘Preferred Reporting Items for Systematic Reviews’ (PRISMA) extension statement for reporting systematic reviews incorporating network meta-analyses of healthcare interventions [10]. In addition, this study was carried out as per the Cochrane systematic review guidelines [11]. This study was prospectively registered in 13 of March 2024 with the International Prospective Register of Systematic Reviews and Meta-Analysis (PROSPERO ID: CRD42024519898).

### 2.2. Inclusion and Exclusion Criteria

For inclusion in this analysis, studies had to meet the following inclusion criteria: retrospective studies, prospective studies, randomized clinical trials or case controls published in English, and no restriction on the publication date. Both genders, aged from 18 years old to 90 years old, and traumatic patients who needed or received whole blood transfusion were included. Studies failing to meet these strict eligibility criteria were excluded. The reasons for study exclusion were as follows: irrelevant intervention, improper method (reported a meta-analysis/systematic review, economic analysis, narrative review, review articles, editorial), other different interventions (packed red blood cells), military settings, pediatric population, animal studies, inadequate data reporting, wrong study design, repetitive publications, language limitation, and non-availability of the full text.

The primary outcomes of this systematic review were barriers and obstacles (adverse events/transfusion reactions, issues with cost, supply/demand and handling of Whole Blood that limit its use, cultural beliefs). Secondary outcomes were mortality rate and other barriers.

### 2.3. Information Sources and Search Strategy

A systematic review and meta-analysis of the literature was performed by a broad electronic search through PubMed, Google Scholar, ScienceDirect, Web of Science, and ProQuest databases for all relevant articles published in English in the last ten years (2013–2023). Two independent reviewers initially searched each database for relevant titles. The keywords used were whole blood transfusion and civilians.

### 2.4. The Selection Process, Data Collection Process, and Analysis

All articles from the primary search were imported to Rayyan [12] for duplication removal and independently screened by three authors (Z.A., E.M, R.S.) based on title and abstract. Two authors reviewed the full text of all studies (L.Z., W.M.). The retrieved studies were reviewed to ensure the inclusion criteria were met for the primary outcome at a minimum, with discordances in opinion resolved through consultation with the third reviewer (L.Z.) by reading the title and abstract and applying the inclusion/exclusion criteria. Disagreements at any step of the screening process were handled through debate and consensus among all authors. Two authors (N.M., A.H.) manually independently extracted the data. An Excel sheet [Version 16.87] was used that included patients’ data and demographics (age, gender), study characteristics (primary author’s name, year of publication, country, study design, sample size), barriers (cultural beliefs, transfusion reactions, adverse events, electrolyte imbalance coagulopathy, issues with cost, mortality)

Tables demonstrated the included studies’ characteristics and results. A PRISMA chart flow figure shows the process of study selection. Moreover, forest plots illustrated each outcome. The meta-analysis was performed by random effect models using RStudio with the Meta Package [13].

### 2.5. Data Items

The combinations and terms used were as follows: (Fresh whole blood OR Transfusion OR Low-titer whole blood OR Massive bleeding OR Critical hemorrhage OR Acute bleeding AND Trauma OR Injury OR Damage control AND Civilian). The title, abstract, or medical subject heading identified the search terms.

### 2.6. Quality Assessment

**Figure 1:**
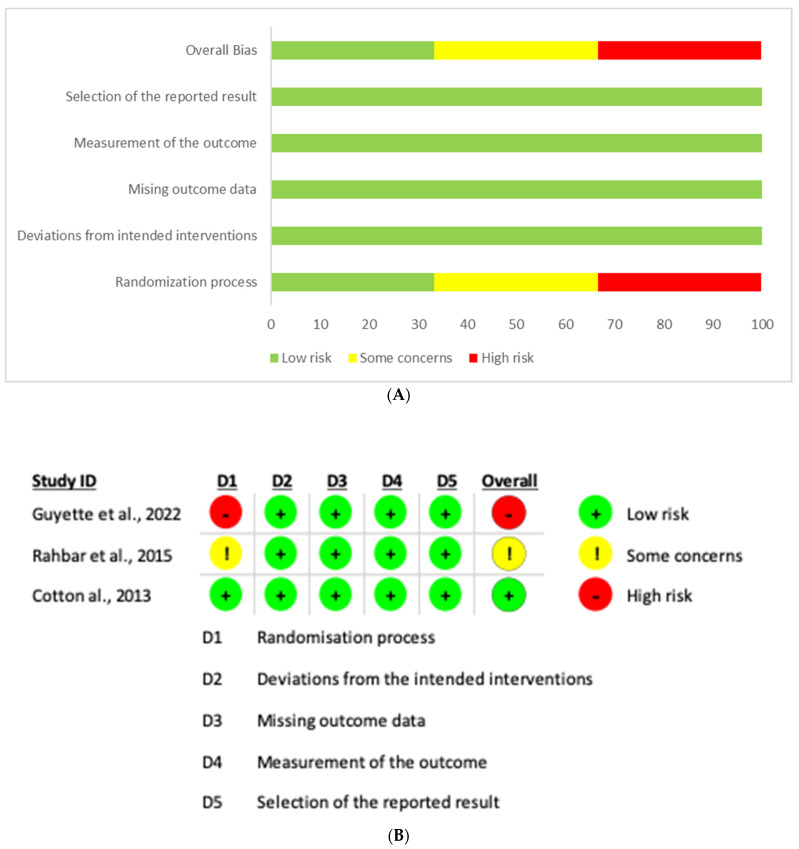
(**A**) Quality assessment summary of the included RCTs. (**B**) Graph for quality assessment of the included RCTs [14,15,16].

Three RCTs were included in this systematic review and meta-analysis. The two authors assessed the risk of bias for each selected article independently based on a template from the Cochrane Handbook Systematic Review of Interventions [11]. The items of assessment included random sequence generation, blinding of outcome assessment, allocation concealment, incomplete outcome data, evaluation of reporting bias, and other types of bias. Each item was assessed using three grades, including low risk, unclear risk, and high risk. Any disagreements were discussed by the two senior authors until consensus was reached. The summary and graph of quality assessment are shown in Figure 1A,B, respectively.

Five of the included cohorts showed good quality since they fulfilled most of the domains of the Newcastle–Ottawa Scale [17]. A quality assessment summary of Cohorts is shown in Table 1.

### 2.7. Data Analysis

Data were analyzed with the Open Meta-Analyst Software Windows 8 (64-bit) (built 04/06/2015) [26]. We calculated the changes in laboratory and clinical parameters following the formula presented in the Cochrane Handbook for Systematic Reviews and Meta-analysis [11]. We used mean or proportion and 95% confidence intervals in the analysis, and the results were considered significant when *p* values were less than 0.05. Heterogeneity was assessed using the Cochrane Q test and I^2^ statistical test, and the results were considered heterogeneous when the *p* value was less than 0.1.

## 3. Results

**Figure 2:**
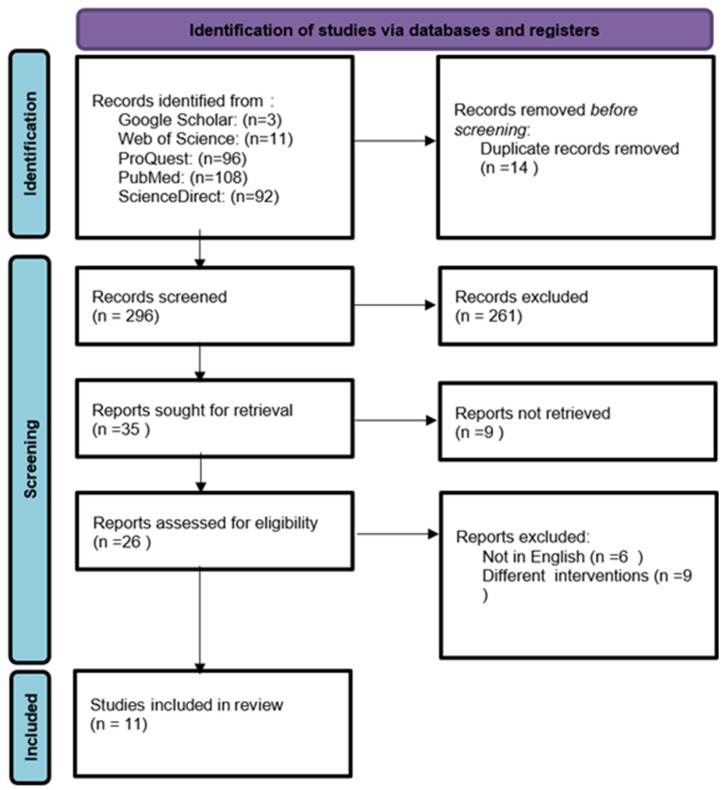
The PRISMA study flow diagram.

The previously mentioned search strategy retrieved a total of 310 articles. After duplicate removal, 296 articles were screened first by title, abstract, and full text. Finally, 11 studies met the inclusion criteria and were included in the systematic review and meta-analysis. The PRISMA study flow diagram is shown in Figure 2. Most of the studies were conducted in cohorts in the United States. The total sample size of the included trauma victims is 1500. The mean age of the patients ranged from 24 ± 38.42 to 49.67 ± 48.2. Males represented about 87.5% of the included sample. The mechanism of injury was mainly blunt trauma. Table 2 shows a summary of the characteristics of the included studies.

### 3.1. Mortality

#### 3.1.1. Six-Hour Mortality

**Figure 3:**
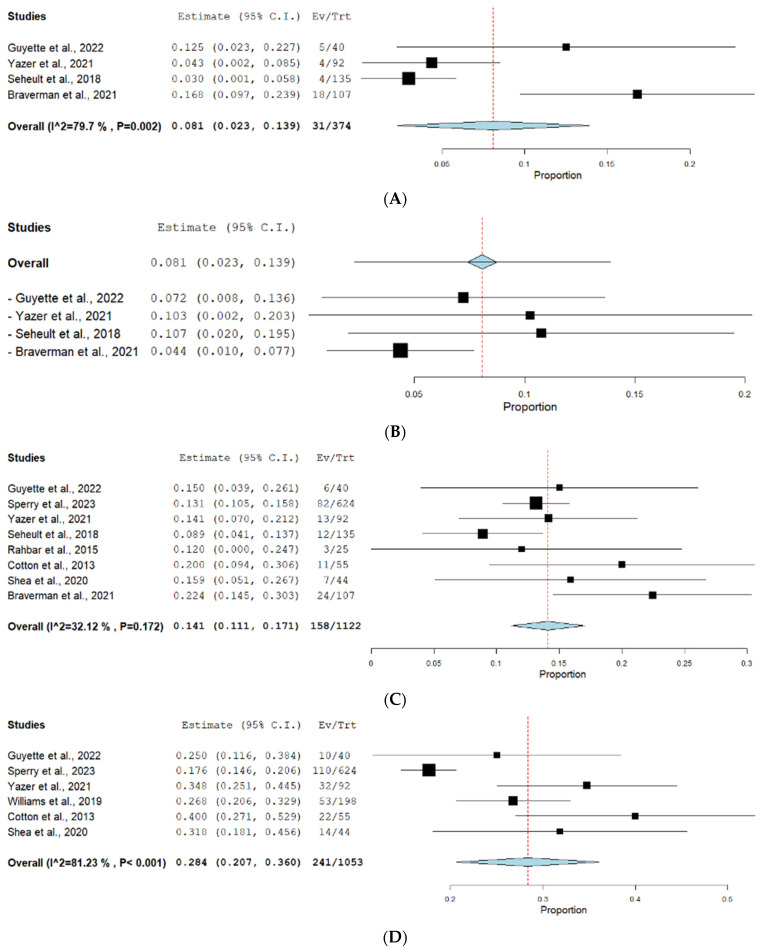

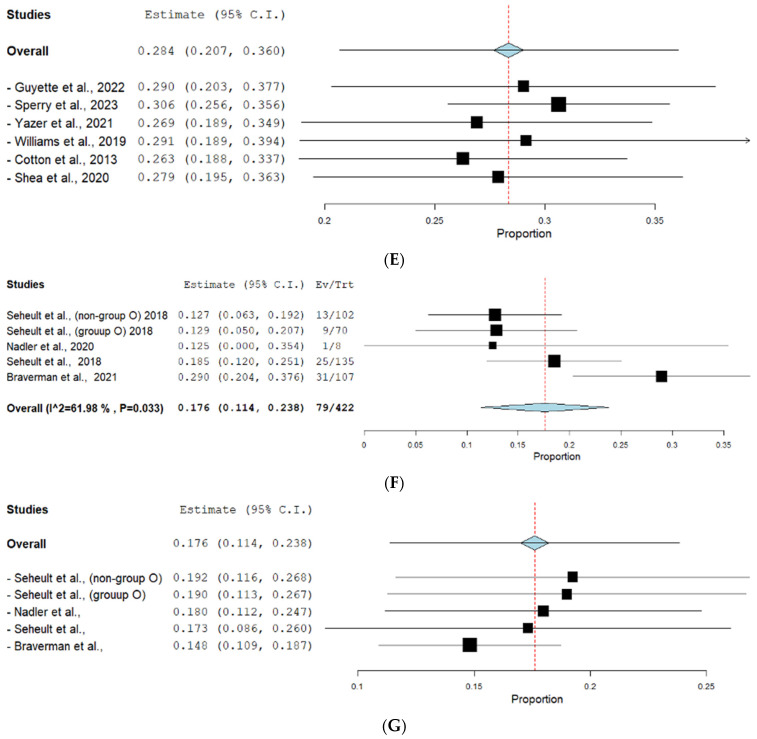
(**A**) 6 h mortality outcome forest plot. (**B**) Leave-one-out test for 6 h mortality outcome. (**C**) 24 h mortality outcome forest plot. (**D**) Delayed mortality outcome forest plot. Leave-one-out test for delayed mortality outcome. (**E**) In-hospital mortality. (**F**) In-hospital mortality outcome forest plot. (**G**) Leave-one-out test for in-hospital mortality outcome. Prism refers to the net result of all the included studies while each square refers to the result of each of the included studies. The red line refers to the net proportion of all the included studies in a single analysis.

Four studies reported the outcome of 6 h mortality. The prevalence of 6 h mortality was 12.15% under the random effect model (0.081, 95% CI [0.023, 0.139]). The pooled studies showed substantial heterogeneity (*p* = 0.002, I^2^ = 79.7%). The forest plot for this outcome is shown in Figure 3A. A leave-one-out test was conducted, as shown in Figure 3B, which suggests that Braverman et al., 2021 [25] is the major source of heterogeneity.

#### 3.1.2. 24 h Mortality

Eight studies reported the rate of 24 h mortality. The prevalence of 24 h mortality was 14.08% under the fixed effect model (0.141, 95% CI [0.111, 0.171]). The pooled studies were homogenous (*p* = 0.172, I^2^ = 32.12%). The forest plot for this outcome is shown in Figure 3C.

#### 3.1.3. Delayed Mortality (28–30 Days)

Six studies reported the rate of delayed mortality at 28–30 days. The prevalence of delayed mortality was 22.89% under the random effect model (0.284, 95% CI [0.207, 0.360]). The pooled studies were heterogeneous (*p* < 0.001, I^2^ = 81.23%). The forest plot for this outcome is shown in Figure 3D. As shown in Figure 3E, the leave-one-out test suggests that Sperry et al. [18] is the major source of heterogeneity.

A single-arm meta-analysis was conducted for the four studies reporting in-hospital mortality among the WBT group. The overall mortality rate was 18.72%, under the random effect model with a relative risk (0.176, 95% CI [0.114, 0.238]). The pooled studies were heterogeneous (*p* = 0.033, I^2^ = 61.98%). The forest plot for this outcome is shown in Figure 3F. A leave-one-out test was conducted, and it showed that Braveman et al. 2021 [25] is the major source of heterogeneity, as shown in Figure 3G.

### 3.2. Course of the Disease Outcomes

#### 3.2.1. Hospital Length of Stay

**Figure 4:**
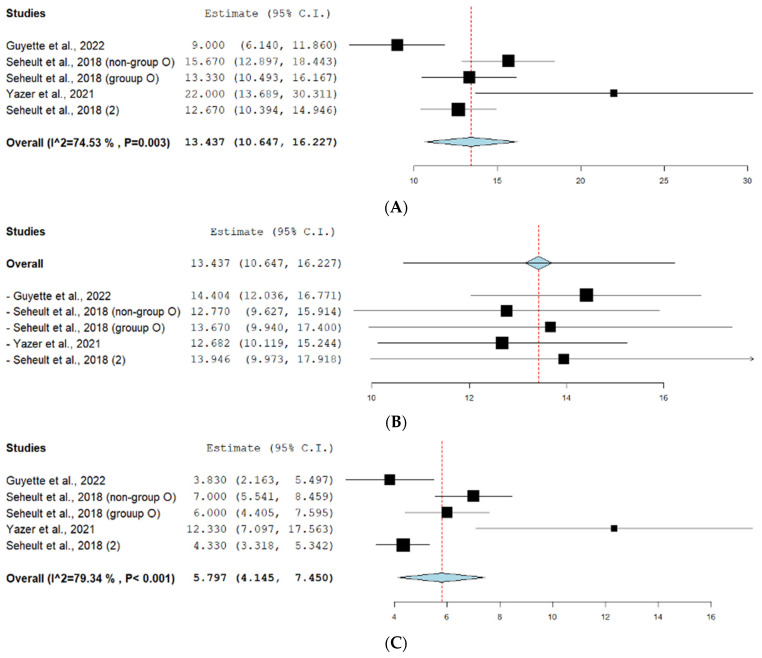

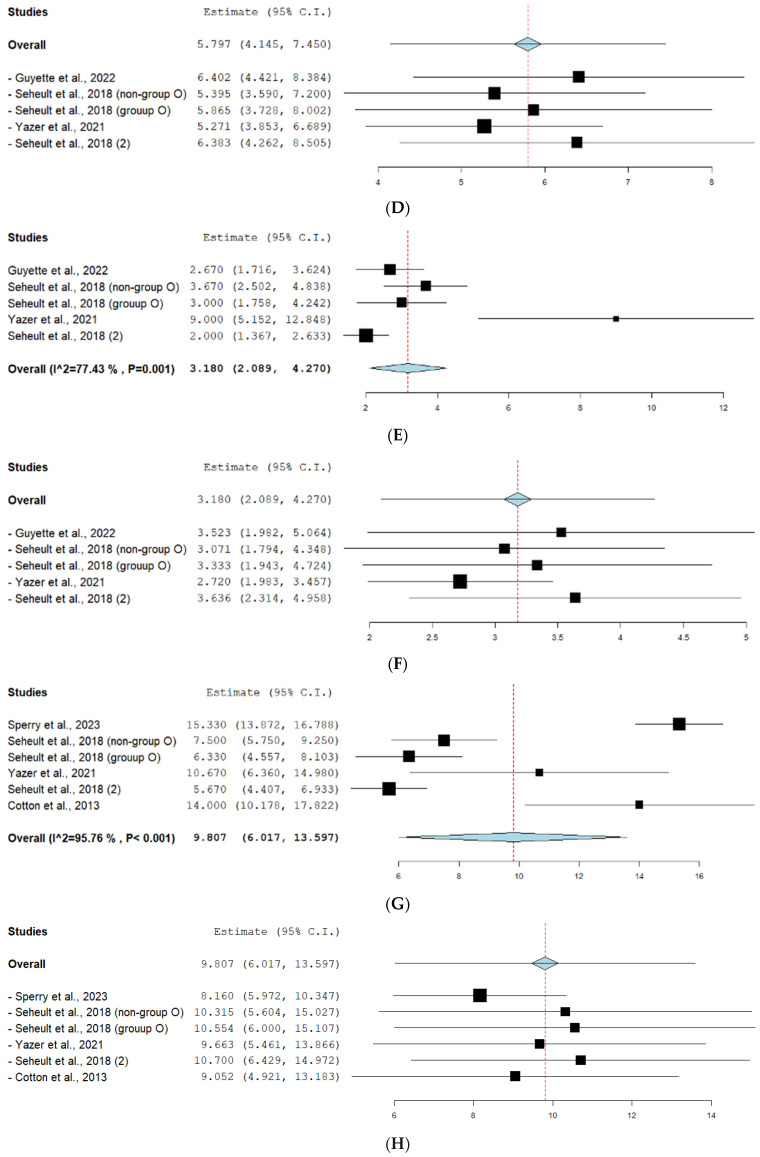

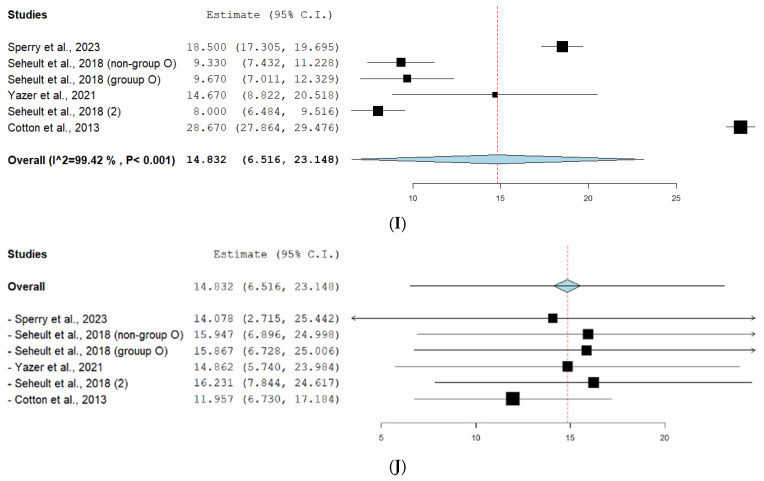
(**A**) Hospital length of stay outcome forest plot. (**B**) Leave-one-out test for hospital length of stay outcome. (**C**) Forest plot for ICU days outcome. (**D**) Leave-one-out test for ICU days outcome. (**E**) Forest plot for days on ventilator outcome. (**F**) Leave-one-out test for ventilator days outcome. (**G**) Forest plot for ICU free days outcome. (**H**) Leave-one-out test for ICU-free days outcome. (**I**) Forest plot for ventilator-free days outcome. (**J**) Forest plot for ventilator-free days outcome after leave-one-out test. Prism refers to the net result of all the included studies while each square refers to the result of each of the included studies. The red line refers to the net proportion of all the included studies in a single analysis.

Five studies reported the mean hospital length of stay. The effect estimate for this outcome under the random effect model was (13.437, 95% CI [10.647, 16.227]). The pooled studies were heterogeneous (*p* = 0.003, I^2^ = 74.53%). The forest plot for this outcome is shown in Figure 4A. As shown in Figure 4B, the leave-one-out test suggests that Guyette et al. [14] is the major source of heterogeneity.

#### 3.2.2. ICU Days

The outcome of ICU days was reported in 4/11 studies. The effect estimate for this outcome under the random effect model was (5.797, 95% CI [4.145, 7.450]). The pooled studies were heterogeneous (*p* < 0.001, I^2^ = 79.34%). The forest plot for this outcome is shown in Figure 4C. A leave-one-out test was performed, as shown in Figure 4D.

#### 3.2.3. Days on Ventilator

The days on ventilator outcome was reported in 4/11 studies. The effect estimate for this outcome under the random effect model was (3.18, 95% CI [2.089, 4.27]). The pooled studies were heterogeneous (*p* = 0.001, I^2^ = 77.43%). The forest plot for this outcome is shown in Figure 4E. A leave-one-out test was conducted, as shown in Figure 4F.

#### 3.2.4. ICU Free Days

The outcome of ICU-free days was reported in 5/11 studies. The effect estimate for this outcome under the random effect model was (9.807, 95% CI [6.017, 13.597]). The pooled studies were heterogeneous (*p* < 0.001, I^2^ = 95.76%). The forest plot for this outcome is shown in Figure 4G. A leave-one-out test was conducted, as shown in Figure 4H.

#### 3.2.5. Ventilator-Free Days

The ventilator-free days outcome was reported in 5/11 studies. The effect estimate for this outcome under the random effect model was (14.832, 95% CI [6.516, 23.148]). The pooled studies were heterogeneous (*p* < 0.001, I^2^ = 99.42%). The forest plot for this outcome is shown in Figure 4I. A leave-one-out test was conducted, as shown in Figure 4J, suggesting that Cotton et al. [24] is the major source of heterogeneity.

### 3.3. Side Effects

#### 3.3.1. Multiorgan Failure

**Figure 5:**
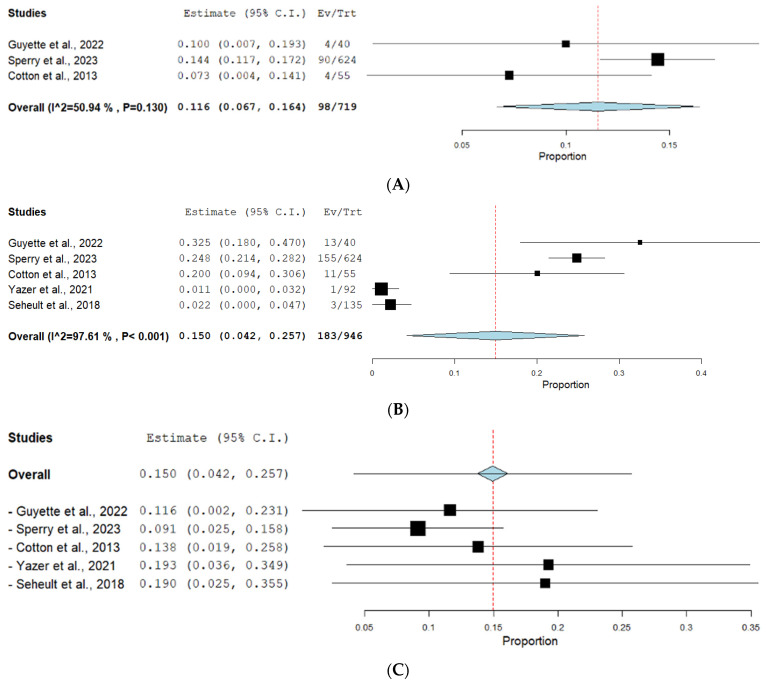
(**A**) Forest plot for multiorgan failure outcome. (**B**) Forest plot for nosocomial infection outcome. (**C**) Leave-one-out test for nosocomial infections outcome.

Multiorgan failure was reported in three studies. The prevalence of multiorgan failure under the fixed effect model was 13.63% (0.116, 95% CI [0.067, 0.164]). The pooled studies were homogenous (*p* = 0.13, I^2^ = 50.94%). The forest plot for this outcome is shown in Figure 5A.

#### 3.3.2. Nosocomial Infections

Nosocomial infections were reported in five studies. The prevalence of nosocomial infections under the random effect model was 19.34% (0.15, 95% CI [0.042, 0.257]). The pooled studies were heterogeneous (*p* < 0.001, I^2^ = 97.61%). The forest plot for this outcome is shown in Figure 5B. A leave-one-out test was conducted, as shown in Figure 5C, which suggests that Sperry et al. [18] is the major source of heterogeneity.

## 4. Discussion

Understanding whole blood transfusion (WBT) in civilian trauma settings is critical due to the evolving setting of trauma care and the potential benefits it could offer in improving patient outcomes [27]. The study aimed to address a gap in the literature on the use of whole blood transfusions (WBTs) in civilian trauma treatment. Traditionally, trauma care has been centered around component therapy, with red blood cells (RBCs), plasma, and platelets administered separately [28]. However, there is growing interest in the potential benefits of WBT, especially in terms of efficiency and patient outcomes during emergency interventions. Despite the potential benefits of whole blood transfusion in trauma settings, such as improved hemostasis and simpler logistics [7,29], many civilian hospitals have been slow to adopt this approach. This is mostly due to logistical, economic, and cultural barriers [30]. Understanding these barriers is critical to creating strategies designed to promote the use of WB transfusions, which may improve outcomes for trauma patients. This systematic review and meta-analysis provides discernment outcomes of the mortality rate and disease outcomes. The key findings showed 6 h, 24 h, and 28–30-day mortality rates of 12.15%, 14.08%, and 22.89%, respectively. Other outcomes included a mean hospital stay of 13.437 days, ICU stay of 5.797 days, ventilator use of 3.18 days, and notable side effects like multiorgan failure (13.63%) and nosocomial infections (19.34%).

### 4.1. Mortality Outcomes

The study reported multiple mortality rates at different periods. The 6 h mortality rate was found to be 12.15 percent, emphasizing the significance of immediate and effective trauma treatment. Immediate trauma treatment is crucial for increasing survival rates and minimizing long-term complications. Early intervention, especially within the “golden hour” after trauma, can have a significant impact on patient outcomes. For instance, Abhilash and Sivanandan underline the need for rapid and effective medical care during the first hour to improve trauma patient survival rates and outcomes [31]. The 24 h mortality rate was somewhat greater, at 14.08%, which may reflect the efficacy of WB transfusion in stabilizing patients immediately but also highlights the necessity for ongoing critical care. The delayed death rate, reported at 28–30 days, was 22.89%, indicating that long-term survival remained difficult even with early management. These findings highlight the crucial role of WB transfusion during the first resuscitation period and the need for comprehensive post-trauma treatment to improve long-term outcomes. A thorough review and meta-analysis of outcomes of transfusion using whole blood, component therapy, or both in adult civilian trauma patients has reported that transfusion with WB + COMP is associated with decreased 24 h mortality compared to COMP, and transfusion with WB is associated with a lower volume of red blood cells transfused at both 6 and 24 h [32].

### 4.2. Disease Outcomes

The study investigated many disease outcomes associated with whole blood (WB) transfusion. Analysis of five studies revealed a mean hospital length of stay of 13.437 days (95% CI [10.647, 16.227]). Additionally, data from four studies indicated an average of 5.797 ICU days (95% CI [4.145, 7.450]). These findings suggest that whole blood transfusion may reduce the need for intensive care compared to component therapy, which often leads to longer ICU stays due to the necessity of multiple transfusions and related complications. This aligns with earlier research conducted by Ngatuvai et al., which found that whole blood transfusion is a safe and effective choice, especially for severe trauma cases [32]. This method reduces the risk of transfusion-induced hypocalcemia compared to blood component therapy containing citrate, leading to shorter hospital stays [31]. Moreover, the mean days on a ventilator, as reported in four studies, was 3.18 days (95% CI [2.089, 4.27]), suggesting that WBT might reduce the need for prolonged mechanical ventilation. This reduction is crucial for patient recovery and in mitigating ventilator-associated complications. Additionally, five studies reported the number of ICU-free days as 9.80795 days (95% CI [6.017, 13.597]), and ventilator-free days as 14.832 days, (95% CI [6.516, 23.148]), both indicating a positive outcome for patients receiving WBT [16,17,18,19,24].

The prevalence of multiorgan failure was reported in three studies as 13.63% (0.116, 95% CI [0.067, 0.164]), suggesting that enhancements in blood transfusion procedures have improved patient outcomes. Prospective observational research concluded that improved 24- and 28-day survival independently correlates with using LTOWB without increasing the organ dysfunction score after 72 h [24]. Nosocomial infections were reported in five studies at a rate of 19.34% (0.15, 95% CI [0.042, 0.257]), highlighting significant morbidity associated with severe trauma [16,17,18,19,24]. These findings underscore the need for improved infection control measures and ongoing monitoring of patients to mitigate these risks. A comprehensive evaluation comparing whole blood transfusion (WBT) to component treatment in trauma resuscitation found no difference in outcomes when whole blood is used [33]. Moreover, a systematic review and meta-analysis of whole blood resuscitation for injured patients demonstrated that whole blood had a consistent benefit over component therapy in terms of decreased blood transfusion and a higher ratio of plasma and platelet to red blood cells in most studies, without significant differences in infectious complications or length of stay. Finally, it should be noted that the type of whole blood used was different among studies [34]. Overall, the disease outcomes observed in this study highlight the potential benefits of WBT in reducing ICU and ventilator dependence, which are critical for recovery. In summary, implementing whole blood transfusion protocols enhances healthcare delivery and patient results and adds to the community’s strength, readiness, and overall welfare. Previous research has highlighted the need for additional high-quality studies regarding whole blood transfusion for females and pediatric age groups [7]. Based on the study’s findings, several recommendations can enhance the adoption and effectiveness of whole blood transfusion (WBT) in civilian trauma care. Hospitals and trauma centers should adopt WBT protocols to reduce ICU stays, ventilator dependence, and overall hospital length of stay. To mitigate the risks of nosocomial infections, stringent infection control protocols should be implemented and monitored. Future research should address the barriers and outcomes of WBT in female and pediatric trauma patients. These recommendations can improve trauma care outcomes and optimize WBT use in civilian settings, enhancing patient recovery and overall healthcare quality.

## 5. Limitations

The studies included in this review only analyzed whole blood transfusion in civilian settings. Second, only whole blood transfusions were used as an intervention. Also, there was heterogeneity among the included studies which might be due to clinical and demographic differences between the patients including age, cause of trauma, and amount of transfused blood.

## 6. Conclusions

This systematic review and meta-analysis aimed to identify the barriers and obstacles to whole blood transfusion for trauma patients in civilian settings. It explored various outcomes of WBT, such as mortality (6 h, 24 h, 28–30 days), length of hospital stay, days in ICU, days on a mechanical ventilator, and side effects of WBT (multiorgan failure and nosocomial infections). Despite the potential benefits of WBT in trauma settings, many civilian hospitals have been slow to adopt this approach.

The findings of this systematic review have demonstrated the potential benefits of WBT in reducing ICU and ventilator dependence compared to component transfusion. However, it showed greater 24 h and 30-day mortality rates in comparison to the 6 h mortality rate, indicating that WBT is effective in early resuscitation and stabilization of trauma patients but with a crucial need for ongoing critical care. Regarding organ dysfunction, three studies reported a low percentage of multiorgan failure, suggesting improved outcomes with enhancement in blood transfusion procedures. In addition, nosocomial infections were reported to be significant in five studies, highlighting the need for improved infection control measures and ongoing monitoring. However, further research is crucial to address the remaining gaps in knowledge to enhance patient recovery and overall healthcare quality. Also, it is recommended to focus on WBT in the pediatric age group, female only group, and other types of trauma injuries and settings.

## Figures and Tables

**Table 1:** Quality assessment of the included cohorts.

	Selection				Comparability	Outcome			Quality Score
Study ID	Representativeness of the Exposed Cohort	Selection of the Non- Exposed Cohort	Ascertainment of Exposure	Demonstration That Outcome of Interest Was not Present at Start of Study	Comparability of Cohorts on the Basis of the Design or Analysis	Assessment of Outcome	Was Follow-Up Long Enough for Outcomes to Occur	Adequacy of Forest Plot for This Outcome of cohorts	
Sperry et al. [18]	*	*	*	*	**	*	*	*	9 Good
Seheult et al. [19]	*		*	*	*	*	*		6 fair
Yazer et al. [20]	*	*	*	*	**	*	*	*	9 Good
Nadler et al. [21]	*		*	*	**	*	*		7 fair
Seheult et al. [22]	*	*	*	*	**	*	*		8 Good
Williams et al. [23]	*	*	*	*	**	*	*	*	9 Good
Shea et al. [24]	*	*	*	*	**	*	*	*	9 Good
Braverman et al. [25]	*	*	*	*	**	*	*		8 Good

*: is the star given to each study if it has a good quality in the corresponding quality domain. The stars are then collected in a score to calculate the overall quality of each study (according to NOS guidelines).

**Table 2:** Summary of the characteristics of the whole blood transfusion group among all the included studies.

Author and Year	Country	Study Design	Sample Size	Age [Median, (IQR)]	Males (*n* %)	Blunt Injury (*n* %)
Guyette et al., 2022 [14]	United States	RCT	40	46 (27–64)	23 (57.5%)	34 (85%)
Sperry et al., 2023 [18]	United States	Prospective Cohort	624	35.0 (26.0–51.0)	546 (87.5%)	252 (40.38%)
Seheult et al., 2018 (non-group O) [19]	United States	Prospective Cohort	102	38 (26–58)	94 (92.16%)	-
Seheult et al., 2018 (group O) [19]			70	41 (26–60)	70 (100%)	-
Yazer et al., 2021 [20]	United States	Retrospective Cohort	92	47 (19–83)	86 (93.48%)	74 (80.43%)
Nadler et al., 2020 [21]	Israel	Retrospective review	8	25.0 (2.0, 45.0)	6 (75%)	8 (100%)
Seheult et al., 2018 [22]	United States	Retrospective Cohort	135	40 (26–61)	129 (95.56%)	109 (80.74%)
Rahbar et al., 2015 [15]	United States	RCT	25	40 (29, 55)	21 (84%)	16 (64%)
Williams et al., 2019 [23]	United States	Retrospective cohort	198	41 (26, 56)	143 (72.22%)	141 (71.2%)
Cotton et al., 2013 [16]	United States	RCT	55	40 (29, 56)	43 (78.2%)	-
Shea et al., 2020 [24]	United States	Prospective Cohort	44	32 (28–32)	35 (79.55%)	12 (27.27%)
Braverman et al., 2021 [25]	United States	Retrospective Cohort	107	32 (24–46)	90 (84.11%)	39 (36.45%)

RCT: randomized controlled trial.

## Data Availability

No datasets were generated or analyzed during the current study.
